# Impact of Health Education on COVID-19 Vaccine Uptake Among Women of Reproductive Age: A Focus on Prepregnant, Pregnant, and Postpartum Populations

**DOI:** 10.7759/cureus.101135

**Published:** 2026-01-09

**Authors:** Aparna Jarathi, Syama Sundar Ayya, Kishore Yadav Jothula

**Affiliations:** 1 Obstetrics and Gynaecology, All India Institute of Medical Sciences, Bibinagar, Hyderabad, IND; 2 Anaesthesiology and Critical Care, All India Institute of Medical Sciences, Bibinagar, Hyderabad, IND; 3 Community and Family Medicine, All India Institute of Medical Sciences, Bibinagar, Hyderabad, IND

**Keywords:** counseling, covid-19 vaccine uptake, individual health, postpartum, pregnant, prepregnant, quasi-experimental design

## Abstract

Introduction: COVID-19 vaccination is essential for protecting reproductive-aged women, yet hesitancy remains high during the prepregnant, pregnant, and postpartum periods. Targeted health education may play a key role in improving vaccine uptake and informed decision-making in these populations.

Methods: A quasi-experimental one-group pre-posttest study was conducted at the All India Institute of Medical Sciences, Bibinagar, India, from February 2022 to June 2022. A total of 149 unvaccinated and partially vaccinated (defined as those who had received only the first dose and had not completed the second dose as per the recommended schedule) women aged 18-29 years were recruited. All enrolled women received targeted health education through structured counseling sessions led by a trained nursing officer to inform and address concerns about the COVID-19 vaccine. Vaccine uptake post-intervention was tracked, and the effectiveness of the counseling was assessed by the number of participants who received the vaccine.

Results: Of the 149 women enrolled, 119 were analyzed, as 30 were lost to follow-up and could not be reached by telephone during the two-month follow-up period to determine their vaccination status. Sixty (50.4%) received the COVID-19 vaccine following the educational intervention. Among partially immunized women, the counseling success rate was 60%, whereas it was significantly lower among previously unvaccinated women (34.1%).

Conclusion: Health education can significantly influence COVID-19 vaccine uptake among reproductive-aged women. However, lower uptake among unvaccinated women suggests that additional strategies are necessary to overcome vaccine hesitancy.

## Introduction

The COVID-19 pandemic has posed unprecedented challenges to global public health, and vaccination has emerged as the cornerstone for reducing disease transmission, severity, and mortality. While mass immunization efforts have been effective in many regions, vaccine hesitancy hinders optimal coverage, particularly among specific vulnerable populations such as women of reproductive age [1]. This population, comprising prepregnant, pregnant, and postpartum women, faces unique concerns and decision-making dynamics that can significantly affect vaccine uptake [2].

Pregnant and postpartum women are known to be at increased risk of severe complications from COVID-19, including hospitalization, intensive care admission, and adverse neonatal outcomes such as preterm birth [3]. Despite these elevated risks and the recommendations issued by the Ministry of Health and Family Welfare (MoHFW), vaccination rates among pregnant individuals remain disproportionately lower than those in the general population, largely due to persistent concerns regarding vaccine safety during pregnancy [4,5]. Moreover, misinformation and sociocultural beliefs can further contribute to vaccine hesitancy in low- and middle-income settings, where reproductive-aged women may have limited access to evidence-based health education.

Health education and structured counseling effectively improve vaccine acceptance across diverse populations. Interventions that are culturally sensitive, phase-specific, and delivered by trusted healthcare providers, such as community health workers and primary care physicians, may significantly enhance vaccine confidence and decision-making, particularly among high-risk groups, including pregnant women.

The primary objective of this study was to assess the effectiveness of individualized counseling sessions led by a trained nursing officer in improving COVID-19 vaccination coverage among young reproductive-aged women, including prepregnant, pregnant, or postpartum women. The secondary objectives were to identify the key barriers to vaccine acceptance in this population and to explore the sociodemographic and behavioral factors influencing their decisions.

## Materials and methods

This study employed a quasi-experimental, one-group pretest-posttest design (O1 X O2) to evaluate the impact of the intervention. After obtaining approval from the Institutional Ethics Committee (AIIMS/BBN/IEC/SEP/2021/105-A), written and verbal informed consent were obtained from all participants. All females aged 18-29 years attending the obstetrics and gynecology outpatient department at the All India Institute of Medical Sciences (AIIMS), Bibinagar, were screened for their COVID-19 vaccination status. Women with a prior history of anaphylaxis or anaphylactoid reactions to any vaccine, or those unwilling to participate, were excluded; however, no participants were excluded at enrollment. A total of 149 unvaccinated and partially vaccinated (defined as those who had received only the first dose and had not completed the second dose as per the recommended schedule) women aged 18-29 years were recruited from the outpatient obstetrics and gynecology clinic between February and April 2022.

All participants were given a questionnaire to collect data on their knowledge, acceptance, and barriers to COVID-19 vaccination (see Appendices). A structured, pre-tested questionnaire comprising closed-ended (Yes/No) questions was developed to assess barriers to COVID-19 and vaccination. The questionnaire was designed and content-validated by three institutional subject experts, and it received approval from the Institutional Ethics Committee. The questionnaire consisted of two sections. Section A outlined the sociodemographic profile. Section B evaluated barriers to COVID-19 vaccine uptake and included domains assessing participants’ knowledge of the COVID-19 virus and vaccines, perceptions and beliefs regarding COVID-19 and vaccination, psychological constraints related to vaccine-associated concerns, and contextual factors, including physical, geographic, social, and political influences (see Appendices).

Each counseling session lasted for about 20 minutes. Based on their responses to the questionnaire, a trained nursing officer provided all the study participants with the necessary information and counseling regarding vaccination (see Appendices). Thus, in-depth interviews were conducted with everyone. Health education was given to everyone separately about the immediate need for vaccination before arrival, and its unpredictable impact on the future waves. Once the patient had given consent for vaccination, they were directed to the immunization clinic to receive vaccination, depending on the availability of COVID vaccines at our institute, or advised to get vaccinated at a nearby state government vaccination center. The study followed a defined pre-test/post-test structure to measure the intervention's impact. The "pre-test" (at enrollment) assessed baseline vaccination status, knowledge gaps, and specific barriers via the structured questionnaire. The "post-test" assessment, conducted two months after the intervention, specifically measured the primary outcome of COVID-19 vaccination uptake. All participants were tracked telephonically by the investigators to determine their vaccination status. The study lasted five months, three months for recruiting patients and two months for follow-up of vaccination status.

The sample size was determined using a census approach, in which all eligible COVID-19 unvaccinated females aged 18-29 years attending the obstetrics outpatient department during the study period were included. The recruitment period was limited to three months in view of the ongoing COVID-19 pandemic, to promote rapid immunization, as a prolonged study duration could delay the potential public health benefit of the intervention. A total of 149 patients who attended the outpatient clinic during this period constituted the study sample.

Statistical analysis

Data were entered into Microsoft Excel (Microsoft Corp., Redmond, WA, USA) and subsequently analyzed using Jamovi statistical software (version 2.6; The Jamovi Project, Sydney, Australia). Continuous variables are presented as mean (standard deviation), and categorical variables are expressed as absolute numbers and percentages. The Shapiro-Wilk test was used to assess the normality of the data. The McNemar test was used to determine the association between counseling and COVID-19 vaccination uptake. A p-value ≤ 0.05 was considered statistically significant.

## Results

A total of 149 reproductive-aged women were enrolled in the study and received one-on-one health education counseling for COVID-19 vaccine uptake. At baseline, 94 women (63.1%) had either taken the first dose or were delaying the second dose, while 55 women (36.9%) had not taken any dose (Table 1).

**Table 1:** Study population characteristics (n=149)

Characteristics (n=149)	n (%)/Mean ± SD
Mean age	23.68 ± 3.8
Delay for the second dose at enrollment	94 (63.1)
Vaccine not taken at enrollment	55 (36.9)
Lost follow-up	30 (20.1)
Pregnant	71 (47.6)
Postpartum	50 (33.5)
Prepregnant	28 (18.7)

Thirty women could not be contacted after the counseling session; hence, the remaining 119 participants were included in the statistical analysis. Comparison of outcomes between unvaccinated and partially vaccinated women after counseling is shown in Table 2.

**Table 2:** Effect of counseling on vaccination status among unvaccinated and partially vaccinated women (McNemar's test)

Pre-counseling Vaccination Status	Post-counseling		P-value
	Vaccinated, n (%)	Deferred Vaccination, n (%)	
Unvaccinated (n=44)	15 (34.1)	29 (65.9)	0.006
Partially vaccinated (n=75)	45 (60)	30 (40)	
Total (n=119)	60 (50.4)	59 (49.6)	

The reasons for failed counseling are included in Table 3.

**Table 3:** Reasons for failed counseling (n=59)

Variable	n (%)
Pregnancy	36 (61)
Nonspecific reasons include misbelief about vaccination (fear of injection, misbelief about vaccine effect on their health status)	13 (22)
Non-availability of vaccines near their home	6 (10.1)
Side effects of the first dose	4 (6.7)

## Discussion

The present study highlights the effectiveness of health education and counseling interventions in increasing COVID-19 vaccination acceptance among women of reproductive age, particularly across the prepregnant, pregnant, and postpartum women. Our findings demonstrate that counseling efforts are successful in 50.4% of the population, underscoring the need for continued innovation and additional strategies to address persistent hesitancy.

Our findings are consistent with the previous literature, which emphasizes the efficacy of targeted educational interventions. Various studies have demonstrated that trust in healthcare providers and direct counseling were strongly associated with vaccine acceptance in pregnant women, particularly when the information addressed vaccine safety and fetal outcomes [6,7]. Similarly, Blakeway et al. reported that vaccine uptake among pregnant women increased significantly after an obstetrician-led education program was implemented [8]. The effectiveness of individualized counseling was found to improve vaccine acceptance [9]. Our study extends these insights by including a wider reproductive demographic and showing a statistically significant improvement post-counseling, even among those initially hesitant.

Previous studies have consistently reported improved vaccine acceptance following educational interventions. For instance, a randomized controlled trial conducted by Abdel-Qader et al. showed that a pharmacist-physician collaborative coaching strategy significantly reduced vaccine hesitancy [10]. In that study, the proportion of participants who received the COVID-19 vaccine increased from 0% to 51.6% within one month post-intervention, demonstrating the critical role of structured health communication in influencing vaccine uptake.

Despite these promising outcomes, our study observed a significant failure rate in counseling pregnant women. Vaccine hesitancy during pregnancy continues to be a considerable barrier, primarily driven by concerns about the safety of the vaccine for both the mother and fetus. Similar findings have been reported in global literature, where pregnant women expressed uncertainty due to the perceived novelty of COVID-19 vaccines, insufficient safety data, and fear of potential adverse fetal outcomes [11].

A notable proportion of pregnant participants in our study cited inadequate motivation from local health workers as a contributing factor in their decision to refuse the vaccine. This finding underscores the urgent need to empower frontline workers, particularly Accredited Social Health Activists (ASHAs), with the necessary knowledge and training to provide accurate, empathetic, and persuasive vaccine counseling. Previous community-based studies have identified ASHA workers as effective facilitators of maternal and child health interventions, particularly in rural and semi-urban settings [1,12].

Additional reasons for vaccine refusal among both pregnant and nonpregnant women included nonspecific fears such as fear of injections, anticipated side effects, and myths regarding vaccine effects on future fertility or general health. Some participants reported mild post-vaccination adverse events like myalgia and fever, which likely contributed to the hesitancy among their peers. Furthermore, logistical challenges, such as the unavailability of the specified vaccine for the second dose, further compounded the barriers to achieving complete vaccination.

After the study, telecounseling was given to 59 unvaccinated women. Of these, 51 (86.6%) expressed a willingness to be vaccinated, especially since many had completed their pregnancies or entered the postpartum period. This reinforces the value of repeated counselling, particularly when timed to coincide with phases of reduced risk perception and increased healthcare engagement.

Despite the positive effect of counseling, overall vaccine coverage in our study population was only 40.2% (of the total enrolled population of 149, including those lost to follow-up), which was substantially lower than the fully vaccinated adult national average of approximately 70% in India during the same period [13]. This disparity highlights a crucial gap in vaccine outreach among reproductive-aged women, especially those who are currently pregnant. Notably, COVID-19 vaccine uptake among pregnant women in our cohort was only 35%, underscoring the urgent need for tailored educational strategies to address their specific concerns.

The WHO Strategic Advisory Group of Experts (SAGE) on Immunisation, which has been guiding global vaccine distribution since 1999, emphasized equity, reciprocity, and prioritizing high-risk groups in its COVID-19 vaccine framework. However, reproductive-aged women, particularly those without comorbidities, were not initially prioritized in vaccination drives despite their vulnerability during pregnancy [14]. The current study highlights the need to reassess these priorities to include this demographic, particularly in future pandemic preparedness policies.

The primary barrier to vaccine acceptance in our study was pregnancy. Among the 59 who did not proceed with vaccination, pregnancy-related concerns accounted for 61% (Table 3). Similar findings were reported in studies by Skjefte et al. and Danchin et al., in which fear of potential harm to the fetus or of pregnancy complications was the leading deterrent, despite clinical evidence supporting vaccine safety [4,15]. Misinformation and misbeliefs, including fear of injections, skepticism about vaccine efficacy, and conspiracy theories, contributed to 22% of refusals in our study. These perceptions align with findings from Khubchandani et al. and Paul et al., who noted that misinformation and mistrust, particularly among women of childbearing age, were significant barriers to vaccine uptake [16,17]. Accessibility issues were also identified, with 10.1% citing a lack of nearby vaccine availability. This structural barrier has been reported in rural settings in both high- and low-income countries. It suggests the need for mobile vaccination units or home-based vaccination drives, especially for postpartum and pregnant women with limited mobility [18].

Interestingly, 51 (86.4%) of the women who remained unvaccinated expressed willingness to receive the vaccine in the future. This indicates that while counseling improved attitudes and reduced hesitancy, structural or personal barriers may have delayed immediate uptake. This delayed behavior change has been similarly documented in studies in which vaccine intention increased after counseling but required sustained follow-up to translate into action [7,19].

The study's strength lies in its real-world setting, including diverse population groups and direct, personalized counseling, which highlights its feasibility for integration into routine antenatal or primary care. Furthermore, the use of a quasi-experimental one-group pre-posttest design without a control group limits the ability to draw definitive causal conclusions, as external factors during the pandemic may have also influenced vaccine uptake. However, the loss to follow-up of 30 participants (20.1%) may limit the generalizability of the findings. Additionally, self-reported willingness does not always predict future behavior, and long-term follow-up would be necessary to confirm whether intentions translate into uptake.

## Conclusions

Health education and personalized counseling significantly improved COVID-19 vaccine uptake among reproductive-aged women, particularly those previously hesitant or delaying the second dose. While the lack of a control group limits definitive causal inferences, the observed increase in vaccination rates highlights the contextual effectiveness of targeted interventions during the pandemic. However, persistent barriers such as pregnancy-related concerns, misinformation, and accessibility highlight the need for multifaceted strategies combining education with improved service delivery. Future public health interventions should leverage trusted healthcare providers, culturally appropriate messages, and follow-up systems to address gaps in vaccine coverage among vulnerable female populations.

## Appendices

**Table 4:** The questionnaire form

Section A: Sociodemographic Profile		
Name		
Age		
Residence		
Socio-economic status		
Have you taken the COVID-19 vaccine? Yes (first dose) Yes (two doses) No		□ □ □
Marital status		Married □ unmarried □
Number of children		
Education		
Health-related educational background		Yes □ No□
Occupation		
Employment		Yes □ No□
Monthly income		
Religion		
Social Category		
Section B: Barriers regarding the COVID-19 virus and vaccine		
Knowledge of the COVID-19 virus and vaccine		
Do you know what does a pandemic mean?		Yes □ No□ Not sure □
Do you know that coronavirus infection and its illness is a pandemic for 2 years?		Yes □ No□ Not sure □
Do you know the symptoms and signs of the illness?		Yes □ No□ Not sure □
Do you know how Coronavirus causes illness?		Yes □ No□ Not sure □
Do you know what are the practices to reduce the transmission of the virus infection?		Yes □ No□ Not sure □
Do you practice hand hygiene? How?		Yes □ No □
Do you wear a mask properly? How?		Yes □ No □
Do you follow physical/social distancing in public places?		Yes □ No□
Did you get tested for the COVID-19 virus anytime for fever or cold?		Yes □ No □
Were you previously infected with the COVID-19 virus?		Yes □ No □
Do you think you won’t be affected by the COVID-19 virus since you are in the young age group?		Yes □ No □ Not sure □
Do you know the COVID-19 virus can cause complications, even in young women, when they are pregnant?		Yes □ No□ Not sure □
If you are pregnant, can you take the vaccination?		Yes □ No□ Not sure □
If you are breastfeeding, can you take the vaccination?		Yes □ No□ Not sure □
If you have any chronic medical diseases like heart disease, epilepsy, asthma, and so on; can you take the vaccination?		Yes □ No□ Not sure □
If you are allergic to any drugs or food items, can you take the vaccination?		Yes □ No □
Are you immunized in childhood?		Yes □ No □ Not sure □
Do you know that vaccination is a simple, safe, and effective way of protecting you against the harmful effects of the Coronavirus?		Yes □ No□ Not sure □
Do you know Covid vaccines prevent you in the first instance from getting sick?		Yes □ No□ Not sure □
Do you know if you are vaccinated against the virus, not only you, the people around you are also protected against the ill effects of the coronavirus?		Yes □ No□ Not sure □
Do you know you can receive the vaccination even after getting infected with the Covid-19 virus?		Yes □ No□ Not sure □
Do you know where the COVID vaccines are given?		Yes □ No□ Not sure □
Is there a COVID vaccine centre near your residence?		Yes □ No□ Not sure □
After receiving the Covid-19 vaccine, do you think it is important to continue to take precautions?		Yes □ No□ Not sure □
Do you know the government has started vaccination of adolescents of age 15-17 years? (Jan 2022)		Yes □ No□
Do you know the government has started booster dose Covid vaccination for eligible populations who completed the 2 doses? (Jan 2022)		Yes □ No□
Perceptions and Beliefs of the COVID-19 virus and vaccine		
Do you think is COVID vaccination is not safe for you?		Yes □ No□ Not sure □
Do you think you don’t need the vaccine because you are young and healthy?		Yes □ No□ Not sure □
Do you think you don’t need the vaccine because you are at home?		Yes □ No□ Not sure □
Do you think you don’t need the vaccine because you do all the right things? wash hands, wear a mask and follow social distancing?		Yes □ No□ Not sure □
Do you prefer Covaxin to Covishield? Why?		Yes □ No□ Not sure □
Are you concerned that the COVID-19 vaccine was rapidly developed and approved?		Yes □ No□ Not sure □
Are you concerned that the COVID-19 vaccine may be faulty or fake?		Yes □ No□ Not sure □
Do you believe that the COVID-19 vaccine is being promoted for the commercial gains of pharmaceutical companies?		Yes □ No□ Not sure □
Psychological constraints with COVID-19 vaccine-related issues		
Are you worried that the vaccine will make you more ill?		Yes □ No□ Not sure □
Are you concerned that the vaccine will not prevent the infection?		Yes □ No□ Not sure □
Are you concerned about the vaccine’s side effects?		Yes □ No□
Are you concerned it may have immediate serious side effects after taking the COVID-19 vaccine?		Yes □ No□ Not sure □
Did any known person of yours have a severe reaction to the COVID vaccination?		Yes □ No□ Not sure □
Are you concerned that the COVID-19 vaccine might have unforeseen future effects?		Yes □ No□ Not sure □
Are you worried that the vaccine will affect future pregnancy or fertility?		Yes □ No□ Not sure □
Are you worried about the route of administration?		Yes □ No□
Will you take the vaccine if it is given other than in parenteral form?		Yes □ No□ Not sure □
Are you concerned about the efficacy of COVID vaccination?		Yes □ No□ Not sure □
Are you concerned about the safety of COVID vaccines which are free of cost in government centres?		Yes □ No□ Not sure □
Are you concerned about the easy availability of your choice of COVID vaccines?		Yes □ No□ Not sure □
Physical, geographic, Social & Political influences (Contextual Factors)		
Is it easy for you if the vaccine is made available nearer to your home?	Yes □ No□	
Which distance will you opt for the centre (0-5 km), (5-10 km), (> 10 km)		
Is it due to the lack of easy availability of COVID-19 vaccine in the centres?	Yes □ No□	
Is it easy for you to take the vaccine at your home?	Yes □ No□	
Is it easy for you to take the vaccine at your workplace?	Yes □ No□	
Will you take the vaccine if it is made mandatory by the Government?	Yes □ No□ Not sure □	
Will you take the vaccine if your family members, relatives or friends suggest it?	Yes □ No□ Not sure □	
Will you go for whole family vaccination?	Yes □ No□ Not sure □	
Will you take the vaccine if you are being given any incentives by the government, other political parties or any other private organisations?	Yes □ No□ Not sure □	
